# Recombinant proteins in therapeutics: haemophilia treatment as an example

**DOI:** 10.1186/1755-7682-1-4

**Published:** 2008-04-28

**Authors:** Antonio Liras

**Affiliations:** 1 Department of Physiology, Faculty of Biology. Complutense University of Madrid, Spain

## Abstract

One of the most spectacular advances in the history of scientific knowledge was the discovery of deoxyribonucleic acid (DNA) by Watson and Crick in 1953. This enabled certain proteins to be prepared in this way for their therapeutic use in clinical practice. Today, in the first decade of the 21st century, hundreds of therapeutic proteins have been produced recombinantly and about 50 of them have been approved for clinical use. Because of the specific procedure used for obtaining these products, which is based on expressing a atherapeutica gene from a fragment of DNA in a cell to produce a functional protein that is free from any human or animal component, they are especially acleana and thus the therapy of choice for many current diseases. The immediate question is: why are recombinant products not used more extensively given their high efficacy and maximum safety? In short, we are faced with an interesting but also unfortunate paradox of pharmacology that greater progress in therapeutic procedures is not always associated with greater introduction of those resources that are safest, for the simple reason that they are more costly.

One of the most spectacular advances in the history of scientific knowledge was the discovery of deoxyribonucleic acid (DNA) by Watson and Crick in 1953 [1]. This discovery made possible what is today known as "modern" biotechnology, in which a fragment of DNA is inserted into a bacterial or eukaryotic cell (animal or plant) to obtain a specific protein – called recombinant – with a specific function. This enabled certain proteins such as insulin, growth hormone, or plasminogen activator to be prepared using this procedure for therapeutic use in clinical practice. Today, in the first decade of the 21^st ^century, hundreds of therapeutic proteins have been produced using recombinant technology, and approximately 50 of them have been approved for clinical use.

However, before this therapeutic approach became a reality, some dramatic iatrogenic consequences had to be assumed, such as the occurrence many years ago of some cases of Creutzfeldt-Jakob disease in children who were treated with growth hormone obtained from post-mortem human pituitary glands. Development of recombinant proteins was also marked by massive and fatal infection with the human immunodeficiency virus (HIV), the cause of AIDS, in the 1980s, and the hepatitis C virus (HCV) in the 1990s.

Putting aside the past, that should be devoted no more time than that required to give a cautionary warning, the present and even more so the future lies in "recombinant medicines". Because of the unique procedure used for obtaining these products, which is based on expressing a "therapeutic" gene from a DNA fragment in a cell to produce a functional protein free from any human or animal component, they are especially "clean", and are therefore the treatment of choice for many current diseases. However, this simple idea, which is not still fully assimilated by the more conservative physicians, was not established in a systematic manner. The first recombinant proteins used in clinical practice, such as somatostatin in 1976, insulin in 1982, erythropoietin in 1986, interferon in 1986, tissue plasminogen activator in 1987, or coagulation factors in 1990, raised a great skepticism because of the fear of what were at the time – but not now – the enormously unknown fields of molecular biology and genetic engineering (Figure 1).

**Figure 1:**
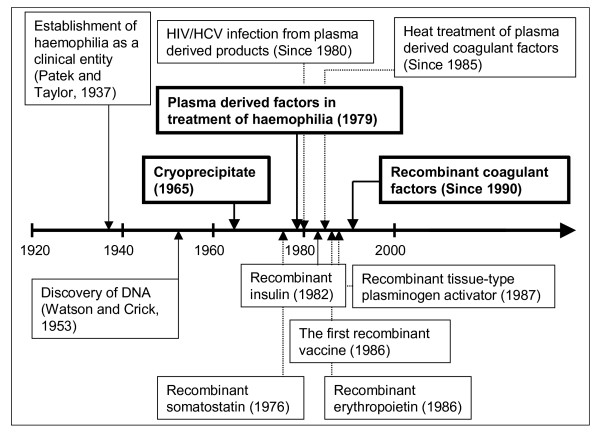
Significant events related to therapeutic recombinant proteins.

Despite this, a large number of biotechnology companies were then created, which resulted in a new concept of pharmaceutical company, the "biotechnology company", and even traditional pharmaceutical companies were reorganized to work in the field of genetic pharmacology. At the same time, the Biotechnology Industry Organization was founded to support the advancement of this sector. Investment continues to increase, and it is estimated that these medicinal products will represent more than 25% of the world pharmaceutical market in the mid or long term.

Many recombinant proteins are today part of standard therapy, and some of them have been used in clinical practice for more than 20 years with an optimal safety and high efficacy and absence of side effects [2]. Others are still in the preliminary study phase, such as those that may be useful in Graves' disease, multiple sclerosis, myasthenia gravis, scleroderma, phenylketonuria, galactosemia, hemoglobinopathies, and other diseases (Table 1).

**Table 1:** Some recombinant proteins used in clinical treatment

**Protein**	**Clinical utility**
Coagulant factors	Haemophilia A and B
DNase I	Cystic fibrosis
Erythropoietin	Anaemia in chronic renal disease
Glucocerebrosidase	Gaucher disease
Growth hormone	Nanism hypophyseal
Insulin	Diabetes
Alpha interferon	Some leukaemias, Kaposi's sarcoma, hepatitis B and C
Gamma-1b interferon	Chronic granulomatous disease
Interleukin-2, -3 and -4	Immunotherapy of cancer
Tissue-type plasminogen activator	Acute myocardial infarction, massive pulmonary embolism
Antibodies for cellular immunotherapy	Neoplasic processes
Vaccines	Influenza, hepatitis A and B
Monoclonal antibodies anti-antibodies	Lupus, rheumatoid arthritis

Recombinant vaccines used in the general population, but also in children, the elderly, and immunocompromised people have unequivocally been shown to be very safe. Recombinant interferon has also been shown to be highly effective and safe for the treatment of hepatitis C. Recombinant peptides are highly useful not only in therapeutics, but also in nutrition as dietary supplements or in cosmetics.

We are now immersed, almost without realizing it, in the "recombinant era", in which we are able to prepare using recombinant techniques, and free from viruses and prions, our own and most abundant protein, albumin, which was previously obtained from human plasma [3].

If the plasma-derived products or human or animal tissues are compared to recombinant products using pharmacological efficacy criteria, no significant differences are found. However, when the criterion used is patient safety, the situation is clearly different. For instance, recombinant products have not caused any serious or even mild adverse events in their 17 years of clinical use for the treatment of haemophilia (1990–2008), and even less adverse events are to be expected with the new third-generation recombinant products, that are completely free from human or animal proteins [4,5]. By contrast, blood plasma-derived products, since 1965, have caused thousands of deaths in hemophilic patients worldwide due to HIV and HCV infection, and will continue to cause deaths because of the side effects of anti-HIV drugs, particularly from cardiovascular and renal disease, and from cirrhosis and hepatocarcinoma in the 60% of patients who do not respond to current treatment for hepatitis C virus. No patient has acquired any significant disease-causing pathogen from a plasma-derived commercial therapeutic since 1987 due to advances in plasma screening, purification and pathogen inactivation technology. Also in the current era of haemophilia care, the most significant complication remains inhibitor development. To this respect there is a lot of controversy [6,7].

Although plasma-derived products of human origin are highly safe as regards lipid envelope viruses such as HIV, HCV, and HBV, they are not free from the well known recently emerging risks resulting from the profound social changes occurring in the world. Such risks include transmission of new diseases, among which particular mention should be made of prion diseases such as the variant Creutzfeldt-Jakob disease (vCJD), or "mad cow disease" [8], which is transmitted through blood [9]. There is also a risk of transmission of the so-called emerging diseases which cause epidemics or pandemics – by crossing the "interspecies" barrier – in populations that were previously unaffected, or by other viruses that may be resistant to viral inactivation processes [10]. At present a lot of measures are been adopted in the selection of blood donors, the use of blood (leucodepletion) and in the plasma-derived products obtention.

There is therefore an almost universal consensus in recommending use of recombinant products. Thus, in Europe, their use is recommended in the "European Guidelines for Recombinant Therapies" [11], and several specific countries, such as the United Kingdom [12], Australia [13], or Spain [14], also advocate their use. Emerging pathogens in haemophilia have contributed to the evolution and adoption of recombinant products (particularly the 3rd-generation products) [15].

The immediate question is: why are not recombinant products more widely used, given their high efficacy and maximum safety? It is difficult to answer this question if we do not go beyond the Hippocratic Medicine we have been taught [16]. It could be for conservative reasons, derived from fear of the unknown, but reasons may also be merely economic or related to "solidarity" in sharing the cost of treatment. It should be remembered that recombinant technology implies higher cost (80 percent higher) from both the treatment itself and the investment for pharmaceutical companies or public administrations that try to implement healthcare policies with an equitable sharing of budgets, though they may sometimes not be in tune with some physicians or with patients themselves. Finally, we cannot rule out the existence of potential conflicts of interest regarding specific products from certain companies in collusion with the physicians who prescribe them.

In any case, clinical practice, which has always involved certain risks, becomes more complicated now that safer and more effective, but also more costly, products are available. In this scenario, the clinician who manages and tries to cure patients is faced with the dilemma of prescribing, sometimes within a very limited range of choices, the safest and most effective pharmacological product with the best safety/efficacy/cost ratio.

In short, we are faced with the interesting but also unfortunate paradox of pharmacology – avoidable for some, unsolvable for others – that a greater progress in therapeutic procedures is not always associated with a greater implementation of those safer resources for the simple reason that they are more expensive. Are we forgetting that patient safety should take precedence over cost or any other underlying reason?
